# Impact of ERK5 on the Hallmarks of Cancer

**DOI:** 10.3390/ijms20061426

**Published:** 2019-03-21

**Authors:** Barbara Stecca, Elisabetta Rovida

**Affiliations:** 1 Tumor Cell Biology Unit–Core Research Laboratory, Institute for Cancer Research, Prevention and Clinical Network (ISPRO), V. le Pieraccini 6, 50139 Florence, Italy; 2 Department of Experimental and Clinical Biomedical Sciences “Mario Serio”, University of Florence, Viale G. B. Morgagni 50, 50134 Florence, Italy

**Keywords:** ERK5, BMK1, MAPK7, MEK5, cancer, cell proliferation, invasion, metastasis, apoptosis, targeted therapy

## Abstract

Extracellular signal-regulated kinase 5 (ERK5) belongs to the mitogen-activated protein kinase (MAPK) family that consists of highly conserved enzymes expressed in all eukaryotic cells and elicits several biological responses, including cell survival, proliferation, migration, and differentiation. In recent years, accumulating lines of evidence point to a relevant role of ERK5 in the onset and progression of several types of cancer. In particular, it has been reported that ERK5 is a key signaling molecule involved in almost all the biological features of cancer cells so that its targeting is emerging as a promising strategy to suppress tumor growth and spreading. Based on that, in this review, we pinpoint the hallmark-specific role of ERK5 in cancer in order to identify biological features that will potentially benefit from ERK5 targeting.

## 1. Introduction

Extracellular signal-regulated kinase 5 (ERK5), the last mitogen-activated protein kinase (MAPK) family member discovered, is activated by the upstream kinase MEK5 in response to growth factors and stresses. While other MAPK members, such as ERK1/2, have well-established roles in tumorigenesis, the less-studied MEK5–ERK5 pathway has emerged as a pivotal player in cancer research only in the last few years. Accumulating evidence in preclinical models indicates the benefits of using MEK5–ERK5 inhibitory strategies for the treatment of human cancers and the contribution of MEK5–ERK5 signaling to therapy resistance. In this review, we summarize recent advances in the influence of ERK5 signaling on the 10 hallmarks of cancer drawn up by Hanahan and Weinberg [1].

## 2. The ERK5 Signaling Pathway

**Figure 1:**
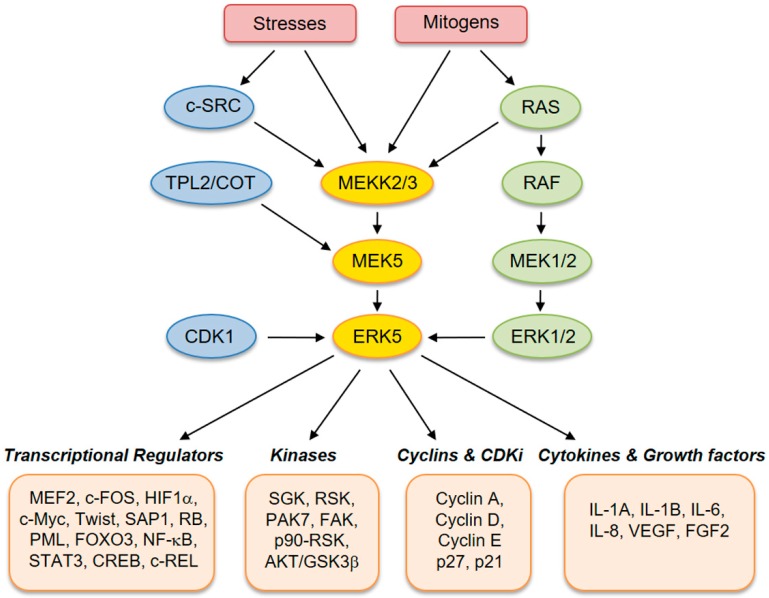
Schematic representation of the MEK5–extracellular signal-regulated kinase 5 (ERK5) pathway with activators and downstream effectors.

ERK5 (also referred to as big mitogen-activated protein kinase 1, BMK-1) is encoded by the *MAPK7* gene and is a member of the MAPK family. ERK5 is ubiquitously expressed in mammalian tissues and cell types, where it is activated by extracellular stimuli, including several growth factors and cellular stresses [2,3,4,5]. Human ERK5 protein contains 816 amino acids and consists of an N-terminal kinase domain (78–406 aa) and a unique C-terminal tail (410–816 aa), which harbors an autoinhibitory function [6]. The C-terminus also contains a myocyte enhancer factor 2 (MEF-2)-interacting region (440–501 aa) [7], a nuclear localization signal (NLS) (505–539 aa), and a transcriptional activation domain (664–789 aa) [7], which associate with and activate several transcription factors [8]. Activation of ERK5 requires dual phosphorylation of threonine and tyrosine residues within a TEY motif in the activation loop of the kinase domain [9]. At this site, ERK5 can be phosphorylated and activated by MEK5, which has a unique specificity for ERK5. Activation by MEK5 induces an open conformation of ERK5, the exposure of the NLS, and the translocation into the nucleus. The latter event is crucial for the proliferative signals induced by ERK5 [10]. Besides being phosphorylated at the TEY motif, ERK5 is able to phosphorylate its C-terminal tail on serine and threonine residues. These residues at the C-terminus have also been reported to be phosphorylated by CDK1 and/or ERK1/2 [11]. Upstream activators of MEK5–ERK5 are MEKK2 and MEKK3, as well as SRC [12], TPL2/COT, RAS, and AKT [13]. Known substrates for ERK5 are transcription factors, including c-FOS, c-MYC, Sap-1a and MEF2A, C and D, and other kinases, such as RSK and serum/glucocorticoid-regulated kinase (SGK) (Figure 1) [14].

## 3. Sustaining Proliferative Signals

ERK5 plays a well-established role in cell proliferation. Several reports have shown activation of ERK5 in response to several mitogens, including epidermal growth factor (EGF) [15], nerve growth factor [16], fibroblast growth factor (FGF) [17], colony-stimulating factor-1 [18], and platelet-derived growth factor (PDGF) [19]. ERK5 regulates different phases of the cell cycle. For instance, ERK5 mediates G1/S transition by regulating the expression of cyclin D1. Conversely, ERK5 inhibition decreases serum-induced cyclin D1 expression [20]. Furthermore, ERK5 is implicated in G2/M transition and is required for mitotic entry. The induction of G2/M by ERK5 depends on the activation of the transcription factor NF-kB, which in turn upregulates mitosis-promoting genes, such as cyclins B1 and B2 and CDC25B [21,22].

**Figure 2:**
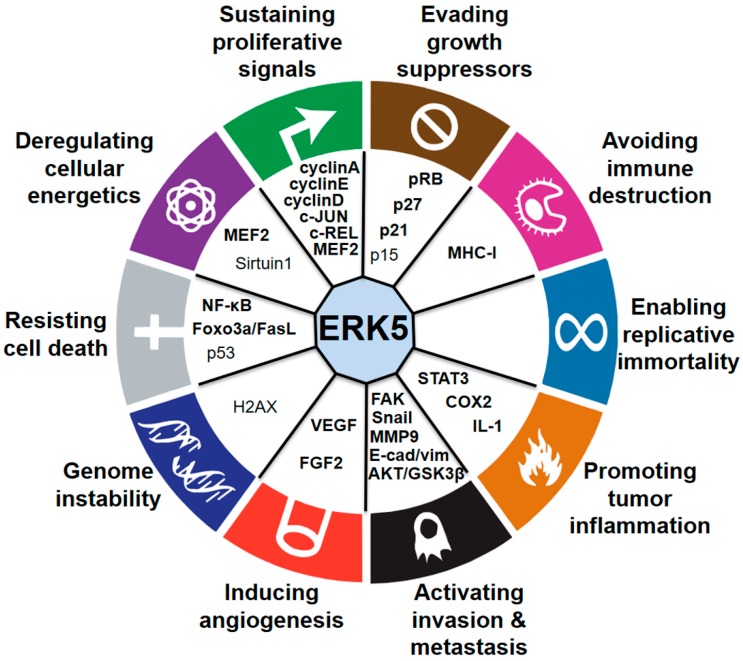
Connections between ERK5 and the hallmarks of cancer. Established (bold) and proposed (nonbold) downstream mediators of ERK5 are indicated. Abbreviations used in the figure are listed in the “Abbreviations” paragraph.

During the last few years, several studies have demonstrated the critical role of MEK5–ERK5 signaling in cancer cell proliferation and tumorigenesis (Figure 2). The role of ERK5 in prostate cancer (PC) proliferation is well established. Human PC displays aberrant expression of ERK5, with significant upregulation of ERK5 protein in high-grade tumors [23]. Increased ERK5 cytoplasmic positivity correlates with Gleason score, bone metastases, and locally advanced disease at diagnosis. Pointing to an important role of nuclear ERK5 in cancer, a subgroup of PC patients shows ERK5 nuclear localization, which correlates with poor disease survival [24]. Functionally, expression of a constitutively active form of MEK5 increases the percentage in the S phase of human PC LNCaP cells, leading to enhanced proliferation in vitro [23]. Along this line, overexpression of ERK5 in PC3 cells increases proliferation in vitro and xenograft growth in vivo [24], whereas ERK5 silencing suppresses PC3 cell proliferation [25]. In addition, EGF-mediated ERK5 activation induces proliferation of RWPE-2 and PC3 cells by promoting entry into the S phase through upregulation of cyclins A and E [26]. Recently, phthalates have been shown to promote PC3 and 22RV1 PC cell proliferation through activation of ERK5 and p38, linking environmental pollution with ERK5 and cancer [27]. The role of microRNA as negative regulators of ERK5 is well documented and implicated in mediating ERK5-dependent PC cell proliferation. MiR-143 inversely correlates with nuclear ERK5 in human PC [28] and interferes with ERK5 signaling to abrogate PC progression in mice [29]. Similarly, overexpression of miR-143 suppresses proliferation of human bladder cancer T24 and Hela cells in vitro and reduces tumor growth of breast cancer (BC) cells in vivo through downregulation of ERK5 [30,31,32].

An early study showed that ERK5 does not affect cell cycle progression of leukemia T cells, but it makes Jurkat T cells more sensitive to tumor necrosis factor α (TNFα). Mechanistically, ERK5 activates NF-kB signaling and promotes nuclear localization and transcriptional activity of p65 [33]. Consistently, ERK5 silencing in EL4 murine leukemia cells blocks tumorigenesis [34]. BCR/ABL-dependent ERK5 expression has been shown to enhance chronic myeloid leukemia (CML) cell survival [6]. A recent report from our group showed that pharmacological and genetic inhibition of the MEK5–ERK5 pathway reduces the growth of CML-patient-derived cells and cell lines in vitro and the number of leukemic cells in a CML mouse model [35]. In the same paper, we identified an important role of ERK5 in CML progenitor/stem cell maintenance and showed that ERK5 targeting in combination with the BCR/ABL inhibitor imatinib reduces the expression of the stem-cell-related factors NANOG, OCT4, and MYC [35,36].

In BC cells, estrogen receptor α (ERα) determines nuclear localization of ERK5, which functions as a coregulator of ERα-dependent gene transcription. ERK5 acts together with the actin remodeling protein CFL1, and upon hormone exposure, both localize to active nuclear transcriptional hubs. This has been proposed to facilitate PAF1 recruitment to the RNA Pol II complex, inducing gene transcription and possibly cell proliferation [37]. In triple-negative BC (TNBC), there is a correlation between high levels of ERK5 and worse prognosis [38]. Genetic and pharmacological inhibition of ERK5 with TG02, a clinical phase multikinase inhibitor that targets ERK5 and CDK 1, 2, and 9 [39], inhibits TNBC proliferation by blocking G1 to G2 cell cycle transition. In addition, TG02 showed significant antitumor activity in a TNBC xenograft model in vivo and increased the activity of chemotherapeutic agents commonly used to treat TNBC [38]. The combination of ERK5 and HSP90 inhibitors is effective in vitro and in vivo in TNBC [40]. In this respect, ERK5 has been reported to be a client of HSP90 [41].

The role of ERK5 in osteosarcoma (OS) cell proliferation is controversial. An early study showed that ERK5 silencing does not affect OS cell proliferation [42]. On the contrary, a recent report indicated that ERK5 is a direct target of miR-143 and that miR-143-dependent negative regulation of ERK5 decreases cell proliferation. Consistently, ERK5 rescues the reduced proliferation induced by miR-143 in OS cells [43].

The role of ERK5 in supporting proliferation of multiple myeloma (MM) cells is well established. An early report showed that ERK5 is expressed in MM cell lines and patient-derived cells, and that this kinase plays a pivotal role in the regulation of MM proliferation and survival. In myeloma cells, ERK5 acts downstream of interleukin (IL) 6, a cytokine that plays an important function in MM cell proliferation and survival, and IL-6-dependent activation of ERK5 occurs through a RAS- and SRC-dependent pathway. MEF2 transcription factors act as downstream mediators of ERK5 in MM cells [44]. A later report from the same group showed that TG02 inhibits the proliferation of several myeloma cell lines. TG02 displayed significant single-agent activity in two MM xenograft models and enhanced the in vivo activity of the proteasome inhibitors bortezomib and lenalidomide [39].

The functional role of ERK5 in the regulation of intestinal homeostasis and tumorigenesis has been recently unraveled. Either enterocyte deletion of ERK1/2 in mice or MEK1/2 pharmacological targeting in vivo led to upregulation of the MEK5–ERK5 pathway, which maintains cell proliferation under physiological conditions. Additionally, pharmacological targeting of both MEK1/2 and ERK5 caused a more effective suppression of human colorectal cancer (CRC) proliferation [45]. Two further studies provided evidence that ERK5 is implicated in regulating human CRC proliferation. The first showed that MEK5 and ERK5 are overexpressed in the majority of human CR adenomas and adenocarcinomas, and that constitutively active MEK5 promotes G1-S and S-G2/M cell cycle transition through NF-kB activation via IkB degradation [46]. The second report showed that in colon cancer patients, high ERK5 expression is associated with poor prognosis, and that constitutive activation of MEK5–ERK5 promotes proliferation of HCT116 and SSW620 colon cancer cells [47]. In contrast with these reports, another study showed that ERK5 is dispensable for proliferation of cancer cells, including CRC cells, displaying *MAPK7* amplification. Specifically, the authors performed pharmacokinetics analyses of the MEK5 inhibitor BIX02189 [48] in a panel of CRC cell lines harboring KRAS G12C/G13D or BRAFV600E mutations. After finding no evidence that the KRAS–BRAF pathway directly activates ERK5, authors showed that, at a concentration where ERK5 activity was almost completely inhibited, BIX02189 had no effect on cancer cell proliferation. Consistently, cancer cells displaying elevated ERK5 expression due to *MAPK7* amplification were insensitive to MEK5 inhibition or ERK5 downregulation by siRNA [49]. Recently, CDK5 has been shown to activate the ERK5–AP-1 (activator protein-1) signaling axis through phosphorylation of ERK5 at Thr732, providing evidence that the CDK5–ERK5–AP-1 axis can promote CRC carcinogenesis [50]. Regarding miR-143 and ERK5 interplay, overexpression of miR-143 impairs human CRC xenograft growth in mice through inhibition of cell proliferation and reduction of ERK5 [51]. Additionally, microRNA replacement therapy for miR-145 in a model of colon carcinoma has been revealed to be efficient in reducing tumor growth in vivo with concomitant repression of ERK5 and c-MYC [52]. In CRC and gastric cancer cells, ERK5 is repressed by the tumor suppressor SATB2, and constitutive activation of ERK5 restores the SATB2-induced inhibition of proliferation [53,54].

In clear cell renal carcinoma (CCRC), the tumor suppressor VHL has been shown to mediate ERK5 degradation through a prolyl-hydroxylation-dependent mechanism. Moreover, there is a strong correlation between a poor prognosis and ERK5 expression in human CCRC samples. In keeping with a possible role of ERK5 in CCRC, downregulation of ERK5 slightly reduces 769-P cell proliferation [55].

ERK5 plays also a relevant role in mesothelioma cell growth. In about one half of the malignant mesothelioma cell lines analyzed, ERK5 activation appeared to be dependent on hepatocyte growth factor (HGF)-mediated activation of phosphoinositide 3-kinase (PI3K) and MEK5 [56]. Interestingly, rocidolite (blue) asbestos activates ERK5 in LP9 cells and silencing of ERK5 decreases proliferation in vitro of mesothelioma cell lines and attenuates tumor growth in vivo in both subcutaneous and intraperitoneal xenografts. Use of doxorubicin and cisplatin in combination with ERK5 inhibitors showed further reduction in tumor weight and volume in vivo. Microarray analysis in HMESO cells suggested that the effects on cell proliferation are likely mediated by decreased expression of p21-activated kinase 7 (PAK7) [57].

In pancreatic ductal adenocarcinoma (PDAC), inhibition of ERK5 with XMD8-92 inhibits AsPC-1 cancer cell proliferation in vitro and xenograft growth in vivo. XMD8-92-treated tumors demonstrated drastic downregulation of doublecortin-like kinase 1 (DCLK1) and several of its downstream targets, including c-MYC, KRAS, NOTCH1, ZEB1, ZEB2, SNAIL, SLUG, VEGFR1, VEGFR2, and pluripotency genes, via upregulation of tumor suppressor miRNAs let-7a, miR-144, miR-200a-c, and miR-143/145 [58].

Pharmacological or genetic inhibition of ERK5 has been shown to suppress proliferation of neuroblastoma cells. Mechanistically, ERK5 is activated by the anaplastic lymphoma kinase (ALK), an important molecular target in neuroblastoma, through PI3K, AKT, and MEKK3–MEK5–ERK5 signaling pathways. ERK5 is involved in the initiation of transcription of MYCN, an oncogene frequently amplified in neuroblastoma. Interestingly, inhibition of ERK5 with XMD8-92 and ALK with crizotinib has been shown to act synergistically to reduce neuroblastoma cell proliferation in vitro and human neuroblastoma xenograft growth in vivo [59].

The role of ERK5 in hepatocellular carcinoma (HCC) is well established. An increase of *MAPK7* copy number has been reported in more than 50% of primary HCC [60]. ERK5 was identified as a gene responsible for HCC tumorigenesis when comparing the proteomic profiles of highly tumorigenic HCC Huh-7.4 cells vs. nontumorigenic Huh-7.5 cells [61]. Knockdown of ERK5 in SNU449 cells was shown to inhibit cell growth by decreasing the mitotic index, implicating for the first time the involvement of ERK5 in the regulation of mitotic entry [60]. These findings were confirmed by our group, showing that ERK5 silencing in HepG2 and Huh-7 HCC cells decreased cell proliferation. Consistently, treatment with the ERK5 inhibitor XMD8-92 slows down HCC cell proliferation, reducing cells in the S phase and increasing the percentage of cells in G0/G1, which are effects associated with decreased cyclin D1. Interestingly, systemic treatment with XMD8-92 or ERK5 silencing reduced HCC xenograft growth. In the same paper, we provided the first evidence in vitro and in vivo that ERK5 inhibition downregulates c-REL, a transcription factor of the NF-kB family relevant for cell proliferation [62].

Our group has recently shown that ERK5 is expressed in human melanomas. Genetic silencing and pharmacological inhibition of the ERK5 pathway reduce growth of melanoma cells and xenografts harboring wild-type (wt) or mutated BRAF (V600E). Mechanistically, oncogenic BRAF positively regulates expression, phosphorylation, nuclear localization, kinase and transcriptional activities of ERK5. From a therapeutic point of view, it is interesting to note that the combination of MEK5 or ERK5 inhibitors with the BRAFV600E inhibitor vemurafenib is more effective than single treatments in decreasing colony formation and proliferation of BRAFV600E melanoma cells and growth of melanoma xenografts [63]. Another report showed that targeting ERK5 impaired drug resistance to combined inhibition of BRAF and MEK1/2 inhibitors in melanoma. Inhibition of ERK5 by shRNA or XMD8-92 diminishes not only the acquirement of resistance to combined inhibition of BRAF and MEK1/2 but also the proliferation of cells resistant to combined BRAF-MEK1/2 inhibitors [64].

Recently, it has been reported that MEK5 acts as an oncogenic driver in lung cancer. Constitutive activation of MEK5 in mice leads to ERK5 phosphorylation and the appearance of lung tumors. The MEK5–ERK5 pathway is also relevant for human lung adenocarcinoma. Indeed, analysis of data from publicly available datasets showed that high levels of combined MEK5 and ERK5 expression significantly associate with poor overall survival in this cancer [65].

## 4. Evading Growth Suppressors

As discussed in detail in the previous paragraph, it has long been known that MEK5–ERK5 signaling regulates cell cycle progression in certain cellular contexts. We were the first to demonstrate that ERK5 inhibition modulates the activation of the cyclin-dependent protein kinase inhibitor (CDKi) p27 in macrophages [18]. Later studies further confirmed that ERK5 suppresses the expression of the CDKi p21, p27, and p15 in normal as well as in cancer cells, thus resulting in the evasion of growth suppression mechanisms [62,63,66,67]. Regarding the regulation of p21 by ERK5, it has been shown that activated ERK5 inhibits PML-dependent induction of p21. Using XMD8-92, it was later shown that ERK5 inhibition blocks tumor cell proliferation in vitro and significantly inhibits tumor growth in vivo, demonstrating the efficacy and tolerability of ERK5 targeting for cancer treatment in preclinical models [68]. Later on, the same group showed that ERK5-mediated phosphorylation of the tumor suppressor PML induces dissociation by MDM2, downregulating the expression of the p53 tumor suppressor [69]. Moreover, based on evidence obtained in MDA-MB-231 breast cancer cells, it was proposed that ERK5 blocks p21 expression via a mechanism that implicates c-MYC-dependent transcriptional regulation of the miR-17-92 cluster [66]. Regarding p27, we provided evidence that increased expression of p27 upon ERK5 inhibition is likely mediated by AKT/FOXO4. Indeed, XMD8-92 treatment resulted in the partial block of G1/S transition, decreased phosphorylation of AKT, and increased the level of p27 and nuclear FOXO4 [62]. Besides regulating p21 expression, inhibition of either MEK5 or ERK5 reduces RB phosphorylation in melanoma cells [63].

Interestingly, a link between ERK5 and the tumor suppressor ataxia telangiectasia mutated (ATM) has been identified. Indeed, in the absence of ERK5, the development of T-cell lymphoma in Atm−/− mice is reduced, revealing a functional interaction between ATM and ERK5 (see also the section “Genome Instability”) [70]. Transforming growth factor β (TGF-β) signaling is considered a suppressor of tumor growth at the initial stages of tumorigenesis. Despite ERK5 is a well-established mediator of TGF-β-elicited epithelial-to-mesenchymal transition (EMT), a clear tumor-promoting feature (see the section “Activating Invasion and Metastasis”), there is no evidence of a possible role of ERK5 in the regulation of TGF-β-elicited growth-suppressive effects.

## 5. Avoiding Immune Destruction

In recent years, several reports have highlighted a link between ERK5 signaling and the ability of tumors to evade the immune system. For instance, in human and mouse leukemic cells, ERK5 silencing downregulates major histocompatibility complex class I (MHC-I) expression at the plasma membrane. Injection of ERK5-silenced tumor cells in syngeneic mice recruits and activates natural killer (NK) cells and induces production of cytokines such as IFNγ and TNFα, which attract new NK cells to the site. This raises an immune response against wild-type tumor cells that attenuates tumorigenesis in vivo [34]. Similarly, restoration of miR-17/20a functions in murine breast cancer and colon cancer cells mediates the inhibition of the MEKK2–MEK5–ERK5 pathway, leading to the downregulation of MHC-I (H-2D) molecules expressed on tumor cells and enhancing the antitumor activity of NK cells in vivo [71].

Deletion of ERK5 in an established PTEN-deficient mouse model of PC increases T-cell infiltration [72]. A transcriptomic analysis revealed upregulation in the prostate of double-knockout mice of the chemokines CCL5 and CXCL10, two potent chemoattractants for T lymphocytes. Consistent with this effect, prostate epithelial and stroma of tumors from double-knockout mice show an increase of a predominantly CD4+ T-cell infiltrate. These data provide a preclinical proof of concept that targeting ERK5 might enhance T-cell infiltrates in PC, with possible implications for exploiting immunotherapy in this cancer type [72].

## 6. Enabling Replicative Immortality

The involvement of ERK5 in replicative immortality in cancer cells has not been reported. However, prolonged treatment of the neuroepithelial neuroblastoma/Ewing sarcoma SK-N-MC cell line with the telomerase inhibitor GRN163 upregulates the expression of miR-143 [73], a well-known negative regulator of ERK5 [29]. Based on that, we might speculate that ERK5 expression is affected by telomere shortening. On the other hand, cellular senescence, characterized by an irreversible arrest in cell proliferation, is among the biological processes that may prevent cellular immortality [74]. No definitive link has been demonstrated showing the role of ERK5 in this biological process. However, microarray data identified ERK5 among the genes responsible for the maintenance, but not onset, of methotrexate-induced cellular senescence in the human colon cancer C85 cell line [75].

## 7. Promoting Tumor Inflammation

ERK5 has been proposed as a mediator of cancer-associated inflammation in epidermal carcinogenesis. In particular, epidermal expression of ERK5 is required to mediate inflammation in the skin [76]. Indeed, inactivation of ERK5 in epidermal keratinocytes has been shown to prevent inflammation-driven tumorigenesis, with evidence that ERK5 is critical for the development of skin SCC, one of the most common types of human nonmelanoma skin cancer. The causal relationship between ERK5 and inflammation is further supported by the demonstration that epidermal ERK5 controls the expression of a specific subset of proinflammatory cytokines, including IL-1A, IL-1B, and COX-2. Importantly, suppression of inflammation by ERK5 deletion in neoplastic keratinocytes reduces tumor burden [76]. This study demonstrated for the first time that ERK5 in epithelial cells is able to remodel the inflammatory microenvironment to support cancer growth. Consistently, it has recently been shown that the ERK5 inhibitor XMD8-92 reduces mesothelioma tumor growth in vivo and inhibits the expression of inflammasome-related genes, such as Caspase-1, IL-1A, IL-1B, HMGB1, and PYCARD [77], although the mechanism of this regulation remains to be determined.

ERK5 has been shown to positively regulate, through its target MEF2C, factors important for monocytic differentiation in human myeloid leukemia cells [78]. Recently, Tournier’s group demonstrated that selective ERK5 ablation in macrophages blocks phosphorylation of signal transducer and activator of transcription 3 (STAT3), a transcription factor crucial for macrophage polarity, impairing the growth of melanoma and carcinoma xenografts. Furthermore, targeting ERK5 in macrophages induced a transcriptional switch in favor of proinflammatory mediators. This study suggests that blocking ERK5 may represent a treatment strategy to reprogram macrophages toward an antitumor state by inhibiting STAT3-induced gene expression [79]. Important clinical implications have emerged from this study. First, the possibility that targeting protumor macrophages via anti-ERK5 therapy appears to be an attractive strategy for cancer treatment. Second, there is the occurrence of a possible synergistic effect between targeted inhibition of ERK5 in macrophages with immune checkpoint inhibitors. The authors also found that tumor-associated macrophages (TAM) lacking ERK5 exhibit a reduced ability to protect melanoma cells against vemurafenib [79]. This effect is in line with the notion that, in addition to contributing to tumorigenesis, TAM play a critical role in tumor-drug resistance and disease relapse after therapy [80].

## 8. Activating Invasion and Metastasis

The active role of the MEK5–ERK5 pathway in supporting cell migration as well as local and distant invasion during cancer progression is well established in different types of cancer. Initial studies showed that in SKOV-3 cells, a highly motile HER2-overexpressing ovary carcinoma cell line, HER2 sustains focal adhesion kinase (FAK) phosphorylation at Ser910 through the activation of ERK5, likely maintaining HER2-driven cell motility [81,82]. The possible involvement of ERK5 in HER-2-mediated migration/invasion has been also reported in the meningioma IOMM-Lee cell line. In these cells, HER-2 silencing determined a reduction of ERK5 expression and phosphorylation (at TEY), and treatment with the ERK5 inhibitor XMD8-92 prevented a HER-2-induced increase of cell invasiveness [83]. Additionally, ERK5 overexpression significantly increases OVCAR-3 ovary cancer cell invasion and migration in vitro, likely due to increased type II collagen expression, while these effects were inhibited by ERK5 silencing [84].

In BC, ERK5 has been initially reported to be a critical target of FAK in cell adhesion signaling and haptotactic motility on vitronectin in MDA-MB-231 cells [85]. It was later showed that ERK5 is a downstream effector of MET-elicited migration in a number of BC cell lines [86], and that in MDA-MB-231 cells, ERK5-dependent HGF/Met-induced migration involves Sam68, an RNA-processing protein [87]. MEKK2 is a well-known upstream activator of the MEK5/ERK5 pathway [13] and MEKK2-mediated activation of ERK5 by EGFR and HER2 is required for metastasis of orthotopic MDA-MB-231 xenografts [88]. Additionally, MEKK2 was reported to mediate fibronectin-induced ERK5 signaling in MDA-MB-231 cells [89]. ERK5 also mediates migration and invasion elicited by EGF or serum in Huh-7 and HepG2 HCC cell lines in vitro [62]. In the same study, we showed that ERK5 mediates hypoxia-driven migration and invasion in vitro.

In HeLa cells, ERK5 is required for EGF-induced invasiveness in vitro, likely involving a MEF2D/DDIAS (DNA-damage-induced apoptosis suppressor)/β-catenin pathway [90]. Using dominant negative and constitutively active forms, as well as small-molecule inhibitors of ERK5 and MEK5, it was revealed that hormone-induced activation of ERα determined a subcellular relocalization of ERK5 within the nucleus of MCF7 cells, thus allowing ERK5 to participate in ER-dependent regulation of gene transcription. In contrast, in cells lacking ERα, ERK5 localizes at cytoplasmic membrane regions of high actin remodeling and promotes cell motility and invasion of MDA-MB-468 and SKBR3 BC cells. This dual role of ERK5 in ER-negative and -positive BC cells has been proposed by the authors to contribute to the worse prognosis in patients with ER-negative BC [37]. Another study performed with Hs578T and MDA-MB-231 BC cells found that CDC42, a member of the Rho GTPase family, negatively regulates the phosphorylation of ERK5, and that ERK5 expression exhibits an inverse relationship with migration and invasiveness. ERK5 expression was reduced in BC tissues compared with adjacent nontumor mammary tissues. Based on the obtained results, the authors proposed that CDC42 may promote BC cell migration and invasion by inhibiting ERK5 phosphorylation, and that ERK5 expression is inversely correlated with the progression of some breast tumors [91]. The fact that inhibition of ERK5 may result in increased cell migration has been also described by our group in hepatic stellate cells, regardless of the presence or absence of PDGF stimulation, raising the possibility that, despite being a promotility/invasive pathway in the majority of cases, the role of the MEK5–ERK5 pathway may be context dependent [19]. 

Another important contribution demonstrating the relevant role of the MEK5–ERK5 pathway in the invasive phenotype of cancer cells comes from studies performed in PC. MEK5 is overexpressed in resected PC with respect to benign prostatic hypertrophy and its expression is associated with tumor metastases and poor survival outcome. Accordingly, experiments in vitro confirmed that a constitutively active mutant of MEK5 increases the invasive ability of human prostate cancer LNCaP cells. Accordingly, active MEK5 increases the expression of the metalloproteinase (MMP) 9, likely via the involvement of AP-1 [23]. ERK5-dependent expression of MMP9 has also been reported in osteosarcoma U2OS as well as PC3 cells [25,42]. In support of a key role of ERK5 in the invasive behavior of PC, it was later reported that reduced ERK5 signaling significantly inhibits PC3 cell motility and invasiveness. Using PC3 cells stably transfected with ERK5, it was demonstrated that ERK5 signaling significantly promotes the formation of metastasis in an orthotopic PC model. Invadopodia formation was also enhanced by forced ERK5 expression in PC3 cells. Furthermore, in human metastatic PC, nuclear ERK5 immunoreactivity was significantly upregulated when compared with benign prostatic hyperplasia and primary PC [25]. Using PC3 cells, it was also demonstrated that ERK5 is a mediator of the PTK6-p130CAS signaling cascade, which plays an important role in cancer cell migration and invasion [92]. 

ERK5 expression has been associated with the World Health Organization grading of glioma and inversely correlated with patient survival [93]. In glioma cell lines, genetic inhibition of ERK5 decreases HGF-induced cell migration and invasiveness in vitro and reduces the expression of the mesenchymal marker N-cadherin and CD44, which are associated with glioma invasiveness. In the same paper, the authors showed that ERK5 is a target of miR-429, which is reduced in glioma cell lines and specimens due to promoter hypermethylation [93]. Additionally, in glioma cell lines and specimens, ERK5 was found to be a direct target gene of miR-200b-3p, which is downregulated in glioma and essential to miR-200b-induced inhibition of glioma xenograft growth, invasion, and EMT. Accordingly, miR-200b-3p overexpression, besides reducing ERK5 expression, results in the increase of the epithelial marker E-cadherin and the suppression of the mesenchymal marker vimentin [94]. Along this line, cholangiocarcinoma (CCA) samples exhibited a remarkable EMT phenotype and a reduced expression of miR-200b that negatively correlated with that of TGF-β. A regulatory loop involving TGF-β and miR-200b was found to contribute to the maintenance of EMT in CCA via AP-2α and ERK5. The administration of miR-200b promotes tumor regression in vivo and abolishes the maintenance of TGF-β-related EMT in an AP-2α- and ERK5-dependent manner in CCA [95].

*MAPK7* is overexpressed in a subset of OS samples and is associated with a poor prognosis [96]. A causal role of ERK5 in the invasive phenotype of OS cells was demonstrated first in vitro using the U2OS cell line, where ERK5 silencing reduces cell invasion and MMP9 expression [42]. Besides confirming the above results in vitro, another group showed that re-expression of SLUG or MMP-9 in ERK5 knockdown cells restores the invasive phenotypes. Furthermore, compared with the vector-transfected U2OS cells, ERK5 knockdown cells show decreased metastatic potential in experimental lung metastasis in mice [97]. A more recent study performed using the SOSP-M human OS cell line showed that ERK5 overexpression in human OS cells promotes cell migration and invasion, whereas knockdown of ERK5 has the opposite effect [98]. Similar results on migration and invasion were obtained by another study [99].

ERK5 is overexpressed in oral SCC samples with respect to oral mucosa. Moreover, an association of high ERK5 expression with advanced tumor stage and the presence of lymph node metastases has been reported in a subset of patients, linking ERK5 to an invasive phenotype in this cancer [100]. Similarly, although a functional role of ERK5 has not been demonstrated, an observational study with 50 patients diagnosed with CCRC showed that ERK5 expression is increased in 58% of cases and is associated with the presence of metastasis and more advanced stages [101].

A prometastatic role of the activated ERK5 pathway has been documented in CRC. Indeed, MEK5 and ERK5 are overexpressed in human colon adenomas and adenocarcinomas, and increased ERK5 expression correlates with the acquisition of a more invasive and metastatic potential. Furthermore, cells with overactivated ERK5 display increased NF-κB nuclear translocation and transcriptional activity together with increased expression of the mesenchymal marker vimentin. Finally, in mouse xenografts, lymph node metastases were exclusively seen in orthotopically implanted tumors with overactivated MEK5–ERK5 and not in tumors with inhibited MEK5–ERK5 [46]. Another study identified a link between ERK5 and the transcription factor special AT-rich sequence-binding protein 2 (SATB2), which is associated with favorable prognosis in CRC. SATB2 expression suppresses HCT116 cell migration and invasion and reduces ERK5 activity. Conversely, ERK5 constitutive activation restores SATB2-expressing cell migration and invasion [53]. Similar results were obtained in the gastric cancer cell line MGC-803, indicating that ERK5 also stimulates cell migration in gastric cancer [54].

The MEK5–ERK5 pathway is involved in EMT, a biological process that converts stationary epithelial cells into a motile mesenchymal phenotype and the aberrant activation of which is linked to increased local invasiveness and metastasis [102]. In BC cells, MEK5-mediated EMT phenotype depends on functional ERK5 and is associated with upregulation of SNAI2 and ZEB1, two important EMT mediators [103]. Another report showed that miR-143-dependent ERK5 downregulation [104] led to suppression of GSK3β/Snail-induced EMT in BC cells [32]. Accordingly, miR-143-3p is expressed at a lower level in cancer with respect to noncancer breast cells, and its overexpression reduces invasion likely through downregulation of ERK5 [105]. Following STAT3-induced upregulation, MEK5 increases BC cell invasion and EMT [106]. Genetic silencing of ERK5 in the highly tumorigenic and metastasis-prone variant of human MDA-MB-231 (4175 TGL variant) cells reduces circulating tumor cells and lung metastases in orthotopic xenografts. In the same study, ERK5 suppression resulted in increased expression of the epithelial marker CDH1 and the reduction of the mesenchymal marker SERPINE1 [107]. Furthermore, suppression of MEK5–ERK5 signaling completely prevents morphological and molecular changes occurring during TGF-β-induced EMT and forces highly metastatic BC cells into a differentiated epithelial state [108]. In human bladder cancer cell lines (T24 and EJ), ERK5 has been described as a mediator of benzidine-induced EMT [109]. In A549 lung cancer cells, ERK5 does not seem to mediate TGF-β1-induced EMT. In fact, TGF-β1-induced EMT, cell motility, and expression of MMP2 were not blocked by the ERK5 inhibitor XMD8-92 or specific silencing of MEK5 and ERK5. Importantly, in the same study, the MEK5 inhibitor BIX02189 was identified as a TGF-β receptor type I (TβRI) inhibitor and was shown to reduce metastasis formation in a TβRI-derived A549 xenograft mouse model [110]. Another study performed in A549 cells reported that ERK5 supports an epithelial phenotype, thus enhancing mesenchymal-to-epithelial transition rather than EMT. Indeed, ERK5 overexpression augments E-cadherin-mediated cell–cell adhesion, downregulates mesenchymal markers, and decreases cell motility, while ERK5 silencing has the opposite effects. Accordingly, ERK5 depletion promotes metastasis in vivo of BC4T1 cells without affecting the growth of the primary tumors. Mechanistically, ERK5 suppresses the AKT–GSK3β–Snail pathway via DEPTOR in A549 cells [111]. All together, these studies indicate that MEK5–ERK5 signaling plays a predominant role in promoting EMT, although it may elicit different outcomes depending on cellular contexts and inducing stimuli.

## 9. Inducing Angiogenesis

Targeted deletion of ERK5 in mice has revealed that the ERK5 signaling cascade is critical for normal cardiovascular development and vascular integrity [112,113]. Additionally, in vitro studies have revealed that in normal endothelial cells, ERK5 is required for preventing apoptosis, mediating shear-stress signaling, and regulating hypoxia and cell migration [114].

The first evidence that ERK5 regulates tumor angiogenesis was described by Hayashi et al. [115]. Following the establishment of two different xenograft models (B16F10 melanoma and LL/2 Lewis lung carcinoma) in mice, inducible ablation of ERK5 in a mouse model carrying an Mx1-Cre transgene resulted in the regression of the tumor vasculature and a concomitant reduction in tumor volume by 63% and 72%, respectively. Similar results were seen in Matrigel plug assays measuring neovascularization in response to VEGF and FGF2 [115]. Consistently, pharmacological inhibition of ERK5 using XMD8-92 in HeLa and LL/2 xenografts revealed that tumor growth was strongly inhibited and FGF2-mediated angiogenesis was inhibited in Matrigel plugs [68]. Importantly, the same study showed that systemic treatment with the ERK5 inhibitor XMD8-92 did not seem to affect the vasculature of mice, an effect at odds with what had been previously observed following endothelium-specific deletion of ERK5 in mice that resulted in embryonic lethality [113]. Although further studies are needed, targeting ERK5 in order to reduce tumor angiogenesis appears to be a valid anticancer therapeutic strategy.

## 10. Genome Instability

Recent evidence linked ERK5 to genome instability occurring during cancer initiation. Indeed, ERK5 seems to inhibit H2AX phosphorylation in Atm−/− mice that, similar to humans carrying ATM-inactivating mutations, are prone to lymphomagenic tumors, likely contributing to genome instability. The delayed death due to T-cell lymphoma development in Atm−/−; Erk5−/− mice suggests that pharmacological targeting of ERK5 has therapeutic potential for T-cell lymphoma patients carrying inactivating ATM mutations [70].

## 11. Resisting Cell Death

The first evidence that the MEK5–ERK5 pathway is an important mediator in survival signaling and resisting apoptosis in cancer cells came from differential gene expression between apoptotic sensitive (APO+) and apoptotic resistant (APO-) MCF-7 cells. Of the 1186 genes detected, MEK5 was increased by 22-fold, pointing to MEK5–ERK5 as a prosurvival signaling. Moreover, expression of an ERK5 dominant-negative mutant resulted in a dose-dependent increase in cell death and enhanced the sensitivity of MCF-7 cells to treatment-induced cell death [116].

An early study showed that ERK5 inhibits TRAIL-induced cell death. Using a truncated form of ERK5 (ERK5Δ570) that accumulates in the nucleus, the authors showed that nuclear ERK5 favored MEF2-dependent transcriptional activity and inhibited TRAIL-induced cell death in HeLa cells. On the contrary, overexpression of ERK5 in BT474, MCF7, or HeLa cells did not affect TRAIL-induced cell death, indicating the anti-apoptotic function of ERK5 requires nuclear activity [117].

Another important mechanism by which ERK5 resists cell death is through p53. Indeed, ERK5 can suppress p53 function by blocking the interaction between PML and MDM2 [69]. The authors demonstrated that ERK5 deactivation coupled with doxorubicin treatment synergistically enhanced MDM2 nucleolar sequestration and, consequently, promoted PML-mediated p53 upregulation, leading to HeLa and A549 tumor cell apoptosis in vitro and tumor regression in vivo. This study suggests that pharmacological ERK5 inhibition significantly enhances the anticancer capacity of doxorubicin-based chemotherapy [69]. On the other hand, MEK5–ERK5 signaling inhibition increases CRC cell sensitivity to 5-fluorouracil through a p53-dependent mechanism [47]. In addition, ERK5 inhibition enhances cytarabine-induced apoptosis in acute myeloid leukemia (AML) cells, suggesting that ERK5 downregulation by siRNA can trigger apoptosis and overcome drug resistance of leukemia cells [118]. Another report showed that inhibition of ERK5–MEK5 signaling induced apoptosis in leukemic cells expressing the oncogenic mutant FLT3-ITD, which is the most frequent mutation in AML. Using the MEK5 inhibitor BIX02188 [48], the authors showed that activation of AKT downstream of FLT3 was partially dependent on ERK5. Furthermore, inhibition of MEK5–ERK5 induced apoptosis of both FLT3-ITD-transfected Ba/F3 cells as well as the FLT3-ITD-carrying leukemic cell lines MV4-11 and MOLM-13 [119]. In addition, knockdown of cytokine-induced apoptosis inhibitor 1 (CIAPIN1), an apoptosis inhibitor with no homology to apoptosis regulatory molecules of the Bcl-2 or caspase families, sensitizes K562 chronic myeloid leukemia cells to imatinib by regulating apoptosis-associated members via the NF-κB and ERK5 signaling pathway [120]. Inhibition of the MEK5–ERK5–NF-kB pathway induces apoptosis in breast cancer MDA-MB-231 cells by genistein, an isoflavonoid present in soybeans with anti-carcinogenic effects [121]. Finally, the multikinase inhibitor TG02, which also inhibits ERK5 activity, selectively induces apoptosis of patient-derived MM cells with respect to normal hematopoietic cells [39].

An early report showed that overexpression of miR-143 in Jurkat cells induces a significant increase in apoptosis by targeting ERK5, which leads to the promotion of the FOXO3a/FasL positive feedback loop [122]. Accordingly, Wang et al. reported that ERK5 plays a role in Fas-dependent signaling by downregulating the expression of FasL [123]. A recent paper confirmed that miR-143 is able to induce apoptosis in HeLa cells through ERK5 negative regulation [31]. In addition, both miR-143 and miR-145 have been implicated in reducing colon carcinoma growth in vivo and inducing apoptosis through reduction of ERK5 expression in vivo [51,52].

Inhibition of ERK5 is able to suppress cancer stem cells through the induction of apoptosis. Inhibition of ERK5 abrogates the effects of MEK5 activity on the tumorigenicity of A549 lung cancer spheres through upregulation of the apoptosis-associated genes BCL2 interacting protein 3 (BNIP3) and BNIP3 like (BNIP3L), which play a critical role in cell death. The upregulation of BNIP3 and BNIP3L is mediated by hypoxia-inducible factor 1α (HIF1α) [124].

Although in several contexts ERK5 has been shown to play a critical role in promoting survival and escaping cell death, a report identified ERK5 and its direct target MEF2 as important mediators of neurotrophin-3-induced apoptosis in medulloblastoma cells [125], suggesting that the biological action of MEK5–ERK5 can be context dependent. In this respect, ERK5 does not appear to be a survival factor in HCC, melanoma, and CML [35,62,63].

## 12. Deregulating Cellular Energetics

Tumor cells have a tendency to use glucose fermentation to obtain energy instead of mitochondrial oxidative phosphorylation (OXPHOS). In the leukemic T-cell line Jurkat and the murine leukemia L1210 cell line, forced respiration, induced by replacement of glucose with glutamine in the culture medium (OXPHOS medium), leads to increased ERK5 expression and accumulation in mitochondria. Interestingly, genetic inhibition of ERK5 reduces cell viability in OXPHOS medium when compared with control mock-transfected cells, indicating that OXPHOS is impaired in the absence of ERK5 [126]. In the same paper, the authors provided evidence that ERK5 is involved in the control of MHC-I expression, a relevant event for the recognition by CTLs and immune evasion (see the section “Avoiding Immune Destruction”), which occurs upon forced respiration. Based on that, ERK5 and MHC-I expression appears to be linked to cell metabolism. In a subsequent paper, the authors from the same group showed that ERK5 regulates the transcription of the NAD-dependent protein deacetylase sirtuin 1 in leukemic Jurkat T cells upon oxidative stress (i.e., H_2_O_2_ administration). This involves the activation of the transcription factor MEF2 and its binding to the *Sirt1* promoter. Additionally, ERK5 is essential for the antioxidant response and its expression is necessary for optimal survival upon a number of oxidative treatments such as H_2_O_2_ and 2,3-dimethoxy-1,4-naphthoquinone (DMNQ) [127]. Additionally, ERK5 increases the expression of miR-23a–27a–24-2 through MEF2. MiR-23a destabilizes Kelch-like ECH-associated protein 1 (Keap1) mRNA, thus increasing the basal expression of the NRF2-dependent genes NAD(P)H dehydrogenase [quinone] 1 (NQO-1) and Heme oxygenase 1 (HO-1), which are responsible for an antioxidant response in a reactive oxygen species (ROS)-independent manner [128]. In conclusion, few studies have addressed the relevant role of ERK5 in conditions when OXPHOS is used by cancer cells. Interestingly, recent studies have shown that OXPHOS can be also upregulated in certain cancers, including leukemia, and that this can occur even in the face of active glycolysis, thus rendering the targeting of OXPHOS-regulated genes of potential interest for cancer treatment [129]. In this respect, ERK5 is emerging as a regulator of mitochondrial function in nontumor cells [130,131].

## 13. Conclusions

It is clear from the presented literature that ERK5 is extensively studied and significantly linked to several types of cancer. One of the major roles of ERK5 is promoting tumor growth by supporting cell proliferation, as well as evading growth-suppressor-elicited signals directed to prevent damaged cells from surviving. These features are crucial to allow tumor cells to start growing without control. As the tumors establish in their niche, they employ ERK5 signaling to enhance the second most important hallmark of cancer, that is, to facilitate tissue invasion and metastasis. With tumor progression, ERK5 signaling enhances angiogenesis to allow the supply of oxygen and nutrients to cancer cells, and facilitates their spreading. Interestingly, there is increasing evidence on the function of ERK5 in regulating cancer metabolism and promoting immune evasion, which are two additional important features of tumor progression. On the other hand, to the best of our knowledge, no data are available on the possible role of ERK5 in replicative immortality and genome instability, features that certainly need to be investigated in the future.

The rationale for ERK5 targeting in cancer is abundantly supported by the many studies discussed in this review and previously reviewed elsewhere, including the possible role of the MEK5–ERK5 pathway in drug resistance [11,14,132]. Many small-molecule compounds targeting ERK5 (including XMD8-92, JWG-071, and BAY-885) or its main upstream activator MEK5 (BIX02189) have been developed [48,68,133,134,135,136]. Few of them have been tested in vivo with remarkable effects on reducing tumor growth in mouse models, but none of them has been challenged in humans. Clinical studies are available for the multikinase inhibitor TG02 only (referring to what is available from the European Union Clinical Trials Register and the ClinicalTrials.gov register). Two phase I clinical studies in patients with advanced hematological malignancies (AML, ALL, blast crisis, and CML) were completed, but the results have not been disclosed yet. Two additional phase I clinical trials are recruiting patients with anaplastic astrocytoma, glioblastoma, and CRC (ClinicalTrials.gov, assessed on January 2019). Lastly, many ERK5–MEK5 inhibitors are not specific and many off-target effects have been reported, including the inhibition of BRD4 [110,137,138]. However, genetic ablation of ERK5 has extensively showed the relevance of this kinase to the discussed hallmarks of cancer. The fact that the newest most specific ERK5 inhibitors (AX compounds) do not affect cancer cell proliferation [138] raises the questions of whether targeting ERK5 kinase activity is the proper therapeutic approach or, rather, targeting noncatalytic functions of ERK5 has to be pursued. In this respect, mechanisms of nuclear translocation of ERK5 or C-terminus-related biological activities need to be clarified further in order to develop new therapeutic strategies [10,63,139]. On the other hand, the occurrence of yet unidentified compensatory/resistance mechanisms upon inhibition of ERK5 kinase activity may account for the inefficacy of small-molecule compounds targeting ERK5. Based on that, in the near future, efforts need to be directed towards the discovery and development of specific and clinical-grade MEK5–ERK5 inhibitors.

## Abbreviations

ALK	Anaplastic lymphoma kinase
AP-1	Activator protein-1
ATM	Ataxia telangiectasia mutated
BC	Breast cancer
BMK-1	Big mitogen-activated protein kinase 1
BNIP3	BCL2 interacting protein 3
BNIP3L	BNIP3 like
CCA	Cholangiocarcinoma
CCRC	Clear cell renal carcinoma
CDKi	Cyclin-dependent protein kinase inhibitor
CML	Chronic myeloid leukemia
COX2	Cyclooxygenase 2
CRC	Colorectal cancer
DDIAS	DNA-damage-induced apoptosis suppressor
EGF	Epidermal growth factor
E-cad	E-cadherin
EMT	Epithelial-to-mesenchymal transition
ERK5	Extracellular signal-regulated kinase 5
ERα	Estrogen receptor α
FAK	Focal adhesion kinase
FGF	Fibroblast growth factor
GSK3	Glycogen synthase kinase 3
HCC	Hepatocellular carcinoma
HGF	Hepatocyte growth factor
IL	Interleukin
MAPK	Mitogen-activated protein kinase family
MEF-2	Myocyte enhancer factor 2
MHC-I	Major histocompatibility complex class I
MM	Multiple myeloma
MMP	Matrix metalloproteinase
NF-κB	Nuclear factor kappa-light-chain-enhancer of activated B cells
NLS	Nuclear localization signal
OS	Osteosarcoma
OXPHOS	Oxidative phosphorylation
PC	Prostate cancer
PDAC	Pancreatic ductal adenocarcinoma
PDGF	Platelet-derived growth factor
PI3K	PhosphoInositide 3-kinase
SATB2	Special AT-rich sequence-binding protein 2
SGK	Serum/glucocorticoid-regulated kinase
STAT3	Signal transducer and activator of transcription 3
TAM	Tumor-associated macrophages
TGF-β	Transforming growth factor β
TNBC	Triple-negative breast cancer
TNFα	Tumor necrosis factor α
TβRI	TGF-β receptor type I
VEGF	Vascular endothelial growth factor
Vim	Vimentin
